# Preventing Re-Emergence of COVID-19: A National Survey of Public Risk Perceptions and Behavioural Intentions Concerning Travel Plan Among Taiwanese

**DOI:** 10.3389/fpubh.2021.710508

**Published:** 2021-08-23

**Authors:** Li Ping Wong, Ching-Ju Chiu, Haridah Alias, Tony Szu-Hsien Lee, Zhijian Hu, Yulan Lin

**Affiliations:** ^1^Department of Social and Preventive Medicine, Faculty of Medicine, Centre for Epidemiology and Evidence-Based Practice, University of Malaya, Kuala Lumpur, Malaysia; ^2^Department of Epidemiology and Health Statistics, Fujian Provincial Key Laboratory of Environment Factors and Cancer, School of Public Health, Fujian Medical University, Fuzhou, China; ^3^Institute of Gerontology, College of Medicine, National Cheng Kung University (NCKU), Tainan, Taiwan; ^4^Department of Health Promotion and Health Education, National Taiwan Normal University, Taipei, Taiwan

**Keywords:** public health, COVID-19, travel plans, public perception, risk perception

## Abstract

**Background:** The objectives of this study were to investigate risk perceptions and travel intention among the general public in Taiwan during the COVID-19 outbreak.

**Methods:** This study used a cross-sectional online survey to collect data. The questionnaire was disseminated via the social media platform (LINE and Facebook) to the general public.

**Results:** A total of 3,237 complete responses were received, of whom 5.8% (95% CI 5.1–6.7) of the participants reported intent to travel to overseas countries with an apparent community spread and 5.5% (95% CI 4.7–6.3) reported intent to travel to other overseas countries in the next 1 month. A relatively higher proportion (46.5%; 95% CI 44.7–48.2) reported intention for domestic travelling. Participants who viewed travelling to only be risky for older adults or those with medical conditions (OR = 2.19; 95% CI 1.38–3.47) and who perceived that one will not get infected if one takes recommended precautionary measures (OR = 3.12; 95% CI 1.85–5.27) reported higher travelling intention to overseas countries with an apparent community spread.

**Conclusions:** Overall, the findings suggest that risk perceptions were depicted as a strong influence of travel intentions.

## Background

An outbreak of coronavirus disease 2019 (COVID-19), caused by the 2019 novel coronavirus (SARS-CoV-2) which began in Wuhan, Hubei Province, China in early December 2019, has spread throughout mainland China, and has swept into at least 122 countries, with over 140,000 confirmed cases and killed over 5,000 people worldwide as of March 14, 2020 (1). In a meeting on January 30, 2020, the outbreak was declared by the World Health Organization (WHO) a Public Health Emergency of International Concern (PHEIC) as it had spread to 18 countries, with four countries reporting human-to-human transmission (2). The severity and highly contagious nature of COVID-19 have sparked concern worldwide and especially in the regions/countries neighbouring mainland China, such as Taiwan, which is just 81 miles off the coast of mainland China. On March 12, 2020 the WHO declared the spread of the novel coronavirus COVID-19 a pandemic. The first case of COVID-19 in Taiwan was reported on 21 January 2020. On 16 February, the first coronavirus death was reported in Taiwan, and the total number of confirmed cases was 20. The first death, from a locally transmitted case in Taiwan, also marked the fifth fatality outside mainland China (3). Taiwan has been listed by the Centres for Disease Control and Prevention as one of the regions with an apparent community spread (4).

The close proximity of Taiwan to mainland China, coupled with the high frequency of cross-border travel, has placed Taiwan as one of the regions most highly threatened by COVID-19, among the other neighbouring countries in Asia. The Taiwanese government has raised the levels of alert and concern about a potential COVID-19 outbreak in the region during the early onset of the outbreak. Taiwan's government learned from its 2003 Severe Acute Respiratory Syndrome (SARS) experience and established an efficient public health response mechanism for enabling rapid actions to deal with the COVID-19 outbreak (5). Various measures have been undertaken by the Taiwan government to contain the spread of the COVID-19 virus, including travel bans, case identification, and quarantine for those who have recently been to mainland China and who have had close contact with confirmed cases of COVID-19 (4). The quick response and efficient disaster management is believed to have resulted in a relatively lower infection rate in Taiwan relative to other Asian countries such as Singapore, Thailand, Malaysia and Vietnam. As of March 12, 2020, there were only 49 cumulative cases and one death reported (6).

Although of late mainland China, as well as in Taiwan, has seen a slowing down in the COVID-19 infection and death rates, continuity in hazard prevention and control are needed in order to effectively curb the outbreak. As effective COVID-19 treatments and most importantly the vaccine to prevent the novel coronavirus is yet to be available, the society at large should continue to contain the outbreak to prevent its re-emergence. Contact and respiratory precautions are the most crucial measures to prevent the spread of the novel coronavirus, and in this regard, an important means of containing COVID-19 is self-imposed restrictions on travel. In particular, travel to countries with widespread sustained transmission of COVID-19 poses a high risk of infections. There is currently limited information available about travel intention among the general public in Taiwan related to COVID-19. Undoubtedly, travel intention during the pandemic or risky behavioural intention is often mediated by risk perception (7). High risk perception has a negative effect on motivation and intention (8). It is unknown if risk perception influences travel intention among the people in Taiwan during the early phase of the COVID-19 outbreak.

Therefore, the main objective of this study was to determine individual risk perception and travel intention among the general public. As reported in previous studies, the emotional impact of an emergency on a person during an infectious disease outbreak influences individual behaviours (9, 10), therefore this study also investigated the influence of anxiety levels on travel intention. The sources of COVID-19-related travel advice/information were also examined to determine the information-seeking behaviour of the public.

## Materials and Methods

### Study Design and Participants

We commenced a cross-sectional, Web-based anonymous survey using an online questionnaire disseminated to the general public using LINE (a mobile messaging app) and Facebook, the largest social media platform in Taiwan. The snowballing sampling technique was used to recruit the participants. Eligible respondents were Taiwan residents aged 20–70 years. When the respondents completed the survey, they were encouraged to disseminate the survey link to all their contacts with a thank you note at the end. Participants were informed that their participation were voluntary, and consent was implied by the completion of the questionnaire. They were assured that their information will be used anonymously. The survey was conducted from March 4th to 11th, 2020.

### Measures

We developed a questionnaire to assess risk perceptions, anxiety levels, intention to travel, travel-related advice/information source, and demographic characteristics of participants. The questionnaire was developed in English and then translated into Mandarin. Professionals and experts in local language validated the content of the questionnaire, after which it was pilot tested. Risk perception consisted of 3-item questions that assessed participants' risk perception of international and domestic travelling during the current COVID-19 outbreak.

Anxiety symptoms were assessed using a 6-item state version of the State–Trait Anxiety Inventory (STAI-6), adapted from previous studies (11, 12). The STAI-6 has been shown to be highly correlated with the 20-item STAI, and all internal consistency reliabilities are >0.90 (13). The Chinese version of the STAI was used in this survey (14). The participants rated the frequency of experiencing six emotional states, namely being calm, tense, upset, relaxed, content, and worried, in relation to the current COVID-19 outbreak. A 4-point scale was used (1 = not at all, 2 = somewhat, 3 = moderately, 4 = very much). The scores on the three positively worded items were reverse-coded. The total summed scores were prorated (multiplied by 20/6) in order to obtain scores that were comparable with those from the full 20-item STAI (giving a range from 20 to 80) (11). A cut-off score of 44 was used to indicate moderate to severe symptoms (15–17). The reliability of the anxiety symptoms in this study was evaluated by assessing the internal consistency of the items representing the scores. The 6-item state anxiety scale used in this study has good internal reliability (Cronbach alpha 0.82) and correlation with the full STAI is high (*r* = 0.95) (12).

Questions about intention to travel asked participants about their intention to travel (over the next month) to: (1) overseas regions/countries listed by the Centres for Disease Control and Prevention (CDC) with an apparent community spread; (2) other overseas regions/countries not listed by CDC as countries with apparent community spread; and (3) other districts in Taiwan (domestic travel). The list of regions/countries with an apparent community spread is noted in the questionnaire. During the period of data collection, the regions/countries with apparent community spread were mainland China, Japan, South Korea, and Hong Kong.

Participants who indicated intent to travel were further probed whether their reason for travel was essential or non-essential travel. The last section asked participants about the source of their COVID-19-related travel advice/information using multiple-choice answers.

### Ethical Considerations

This study protocol was approved by the Institutional Review Board of National Cheng Kung University Hospital (NCKUH) (No: A-EX-109-013).

### Statistical Analyses

Multivariable logistic regression was used to determine the factors influencing intention to travel to: (1) overseas regions/countries with an apparent community spread; (2) other overseas regions/countries not listed by CDC as regions/countries with apparent community spread; and (3) other districts in Taiwan. Variables that were significant using a chi-square (χ^2^) test in the univariate analyses were selected for multivariate logistic regression analyses and included in the model, using a simultaneous forced-entry method. Odds ratios (OR), 95% confidence intervals (95% CI), and *p* values were calculated for each independent variable. The model fit was assessed using the Hosmer–Lemeshow goodness-of-fit test (18). All statistical analyses were performed using the Statistical Package for the Social Sciences, version 20.0 (IBM Corp., Armonk, NY, USA). A *p* < 0.05 was considered statistically significant.

## Results

A total of 3,237 complete responses were obtained. A summary of the characteristics of the respondents is provided in the first and second columns of Table 1. Most of the responses were from the North (*n* = 1,078, 33.3%), South (*n* = 1,385, 42.8%), and Central (*n* = 719, 22.2%) districts of Taiwan. The majority of the participants were female (71.2%). A great majority had either a university or postgraduate degree (87.1%). Most of the respondents earn an annual income between NT$50,000–100,000 (39.0%) and below NT$ 50,000 (32.7%). The majority perceived their health as very good (40.5%) and good (28.3%).

**Table 1:** Demographic characteristics and the State-Trait Anxiety Inventory (STAI) scores (*N* = 3,237).

	**Frequency (%)**	**Univariate analysis**			**Multivariable analysis**
		**Total STAI-6 score**			**Total STAI-6 score**
		**Score 44–80**	**Score 20–43**	***p***	**Score 44–80** **vs. 20–43** ^**a**^
		**(*N* = 1,798)**	**(*N* = 1,439)**		
**Demographics**					
**Age group (years)**					
18–30	521 (16.1)	264 (50.7)	257 (49.3)		Ref
31–40	820 (25.3)	445 (54.3)	375 (45.7)	0.025	1.01 (0.81–1.27)
41–50	984 (30.4)	554 (56.3)	430 (43.7)		1.11 (0.88–1.38)
>50	912 (28.2)	535 (58.7)	377 (41.3)		1.28 (1.01–1.61)^*^
**Gender**					
Male	932 (28.8)	440 (47.2)	492 (52.8)	*p* < 0.001	Ref
Female	2,305 (71.2)	1,358 (58.9)	947 (41.1)		1.50 (1.28–1.76)^***^
**Highest educational level**					
Secondary school and below	419 (12.9)	255 (60.9)	164 (39.1)	0.020	1.12 (0.90–1.40)
University and above	2,818 (87.1)	1,543 (54.8)	1,275 (45.2)		Ref
**Average household income (NT$)**					
<50,000	1,057 (32.7)	628 (59.4)	429 (40.6)		1.26 (1.04–1.53)^*^
50,000–100,000	1,262 (39.0)	695 (55.1)	567 (44.9)	0.003	1.11 (0.93–1.32)
>100,000	918 (28.4)	475 (51.7)	443 (48.3)		Ref
**Location**					
North district	1,078 (33.3)	611 (56.7)	467 (43.3)		
Central district	719 (22.2)	400 (55.6)	319 (44.4)	0.194	
South district	1,385 (42.8)	750 (54.2)	635 (45.8)		
East district	55 (1.7)	37 (67.3)	18 (32.7)		
**Health characteristics**					
**Perceived health status**					
Excellent	441 (13.6)	188 (42.6)	253 (57.4)		Ref
Very good	1,310 (40.5)	672 (51.3)	638 (48.7)	*p* <0.001	1.35 (1.08–1.68)^**^
Good	916 (28.3)	549 (59.9)	367 (40.1)		1.88 (1.48–2.38)^***^
Fair/Poor	570 (17.6)	389 (68.2)	181 (31.8)		2.61 (2.00–3.41)^***^
**Have chronic diseases**					
No	2,583 (79.8)	1,410 (54.6)	1,173 (45.4)	0.031	Ref
Yes	654 (20.2)	388 (59.3)	266 (40.7)		1.05 (0.87–1.27)

* *p < 0.05,*

** *p < 0.01,*

*** *p < 0.001.*

a *Hosmer-Lemeshow test, chi-square: 9.822, p-value: 0.278; Nagelkerke R^2^: 0.052*.

### Risk Perception

**Figure 1:**
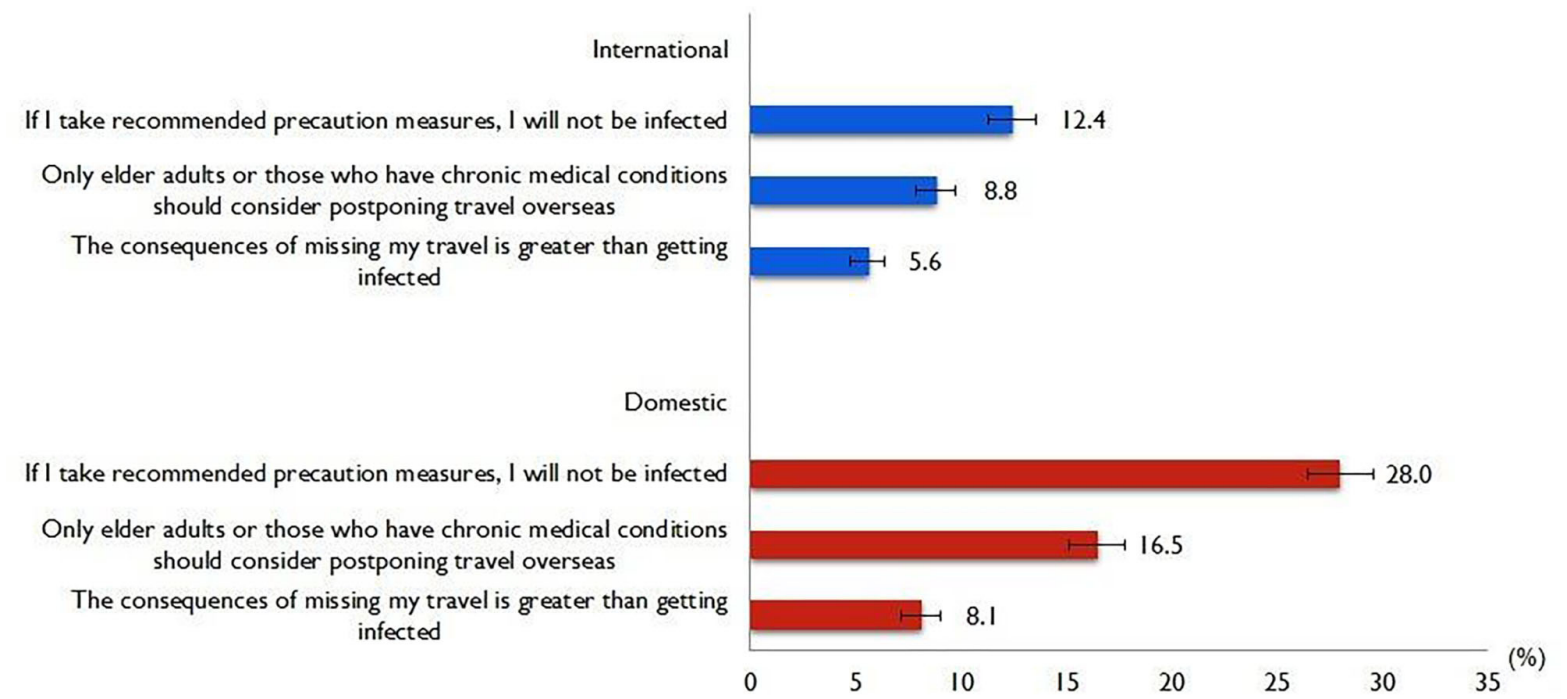
Risk perceptions of international and domestic travels.

Figure 1 shows the responses regarding risk perceptions of international and domestic travelling. A considerable proportion (12.4%, 95% CI 11.3–13.6) viewed that if they take recommended precautionary measures, they will not be infected with COVID-19. A total of 8.8% (95% CI 7.9–9.8) thought that only older adults or those who have a chronic medical condition should consider postponing international travelling and a smaller proportion (5.6%; 95% CI 4.8–6.4) viewed the consequences of missing their international travel as being greater than being infected.

With regard to domestic travelling, a relatively higher proportion (28.0%, 95% CI 26.5–29.6) thought that if they take recommended precautionary measures, they will not be infected with COVID-19. A total of 16.5% (95% CI 15.2–17.8) thought that only older adults or those who have a chronic medical condition should consider postponing domestic travelling and similarly a smaller proportion (8.1%; 95% CI 7.2–9.1) viewed the consequences of missing their domestic travel as being greater than getting infected.

### Anxiety Symptoms

Using a cut-off score of 44 for the STAI score, a total of 55.5% (95% CI 53.8–57.3) of the respondents reported moderate to severe levels of anxiety symptoms (anxiety score of 44–88). The mean anxiety score was 45.2 (*SD* = 10.1). As shown in Table 1, there is a gradual increase in the proportion who reported moderate to severe levels of anxiety symptoms with the increase in age. Females reported a higher likelihood of having moderate to severe levels of anxiety symptoms than males (OR = 1.50, 95% CI 1.28–1.76). The income group below NT$50,000 (OR = 1.26, 95% CI 1.04–1.53) was more likely to report moderate to severe levels of anxiety symptoms than those in the NT$ > 10,000 group. There is a gradual increase in anxiety levels by perceived health status, whereby participants who perceived their health status as fair/poor (OR = 2.61, 95% CI 2.00–3.41), good (OR = 1.88, 95% CI 1.48–2.38), or very good (OR = 1.35, 95% CI 1.08–1.68), were more likely to have a higher proportion of having moderate to severe levels of anxiety symptoms than those who perceived their health status as excellent.

### Intention to Travel

Only 189 (5.8%; 95% CI 5.1–6.7) participants reported intent to travel to overseas regions/countries with an apparent community spread and 178 (5.5%; 95% CI 4.7–6.3) reported intent to travel to other overseas countries in the next month. Among the 189 participants who reported intent to travel to overseas regions/countries with an apparent community spread, a total of 141 (74.6%) reported that the reason for their travel is for a holiday, whereas 35 (18.5%) respondents reported that their travel is work-related. Among those who reported intention to travel to other overseas countries in the next month, the majority (*n* = 108, 60.7%) reported the reason for their travel is for holidays, while 23.6% (*n* = 42) reported work-related travel as their reason.

For domestic travel, a relatively higher proportion (46.5%; 95% CI 44.7–48.2) reported the intention to undergo domestic travelling (to other districts in Taiwan) in the next month. The majority reported travelling reasons being for holidays (*n* = 1,138, 75.7%) followed by visiting friends and relatives (*n* = 190, 12.6%), and a minority associated their travels as being work-related and for education purposes (*n* = 195, 11.6%).

Table 2 shows the multivariable regression analyses of factors associated with travelling intention. None of the demographic characteristics were significantly associated with intention to travel to overseas regions/countries with an apparent community spread. However, participants who reported having a chronic disease have higher odds of intent to travel to overseas regions/countries with an apparent community spread (OR = 1.44, 95% CI 1.03–2.02). Participants who thought that only older adults or those who have chronic medical conditions should consider postponing travel (OR = 2.19; 95% CI 1.38–3.47) and perceived that one will not get infected with COVID-19 if one takes the recommended precautionary measures (OR = 3.12; 95% CI 1.85–5.27), reported a higher intention to travel to overseas regions/countries with an apparent community spread. Likewise, multivariable regression analyses for factors influencing the intention to travel to other overseas countries with no apparent community spread, revealed that those who held the perception that only older adults or those who have chronic medical conditions should consider postponing travel (OR = 1.83; 95% CI 1.15–2.92) and perceived that one will not get infected with COVID-19 if one takes the recommended precautionary measures (OR = 2.09; 95% CI 1.24–3.53) reported higher travelling intention. Additionally, the multivariate regression model also revealed participants of secondary school and below (OR = 2.17; 95% CI 1.49–3.17) reported higher intention to travel to other overseas countries with no apparent community spread than those of university graduates.

**Table 2:** Multivariable regression analyses of factors associated with intention to travel in the next 1 month (*N* = 3,237).

	**Intention to travel**		
	**Regions/countries** **with an apparent** **community spread** **^a^**	**Other countries not** **been listed as countries** **with an apparent** **community spread** **^b^**	**Domestic travel** **(other district/zone** **in Taiwan)** **^c^**
	**Yes (*n* = 189) vs.** **No (*n* = 3,048)**	**Yes (*n* = 178) vs.** **No (*n* = 3,059)**	**Yes (*n* = 1,504) vs.** **No (*n* = 1,733)**
	**OR (95% CI)**	**OR (95% CI)**	**OR (95% CI)**
**Demographics**			
**Age group (years)**			
18–30		Ref	1.33 (1.05–168)^*^
31–40		1.47 (0.87–2.50)	1.55 (1.27–1.90)^***^
41–50		1.05 (0.61–1.78)	1.18 (0.97–1.42)
>50		1.54 (0.92–2.59)	Ref
**Gender**			
Male			1.27 (1.08–1.49)^**^
Female			Ref
**Highest educational level**			
Secondary school and below	1.39 (0.94–2.05)	2.17 (1.49–3.17)^***^	
University and above	Ref	Ref	
**Average household income (NT$)**			
<50,000			Ref
50,000–100,000			1.38 (1.16–1.64)^***^
>100,000			1.45 (1.20–1.76)^***^
**Location**			
North district			
Central district			
South district			
East district			
**Perceived health status**			
Excellent			0.91 (0.69–1.19)
Very good			1.26 (1.02–1.55)^*^
Good			1.14 (0.91–1.42)
Fair/Poor			Ref
**Have chronic diseases**			
No	Ref	Ref	
Yes	1.44 (1.03–2.02)^*^	1.30 (0.91–1.87)	
**Risk perceptions**			
**Only older adults or those who have chronic medical conditions should consider postponing travel**			
Agree	2.19 (1.38–3.47)^**^	1.83 (1.15–2.92)^*^	1.79 (1.42–2.26)^***^
Disagree	Ref	Ref	Ref
**The consequences of missing my travel is greater than getting infected**			
Agree	3.12 (1.85–5.27)^***^	2.09 (1.24–3.53)^*^	1.10 (0.81–1.51)
Disagree	Ref	Ref	Ref
**If I take recommended precaution measures, I will not be infected**			
Agree	0.88 (0.54–1.42)	2.19 (1.43–3.34)	1.10 (0.81–1.51)
Disagree	Ref	Ref	Ref

* *p < 0.05,*

** *p < 0.01,*

*** *p < 0.001.*

a *Hosmer–Lemeshow test, chi-square: 3.128, p-value: 0.372; Nagelkerke R^2^: 0.055.*

b *Hosmer–Lemeshow test, chi-square: 3.661, p-value: 0.722; Nagelkerke R^2^: 0.082.*

c *Hosmer–Lemeshow test, chi-square: 7.965, p-value: 0.437; Nagelkerke R^2^: 0.129*.

Multivariable regression analyses of factors influencing intention for domestic travel showed that by demographics, the younger age group expressed higher intention for domestic travel. Males expressed higher intention for domestic travel than females (OR = 1.27, 95% CI 1.08–1.49). Participants of higher-income groups also expressed higher intention to carry out domestic travelling. Participants who perceived that their health status as very good expressed a higher intention for domestic travel than those of fair/poor (OR = 1.26, 95% CI 1.02–1.55). Participants who thought that only older adults or those who have chronic medical conditions should consider postponing travel (OR = 1.79, 95% CI 1.42–2.26) also expressed higher intention for domestic travel.

**Figure 2:**
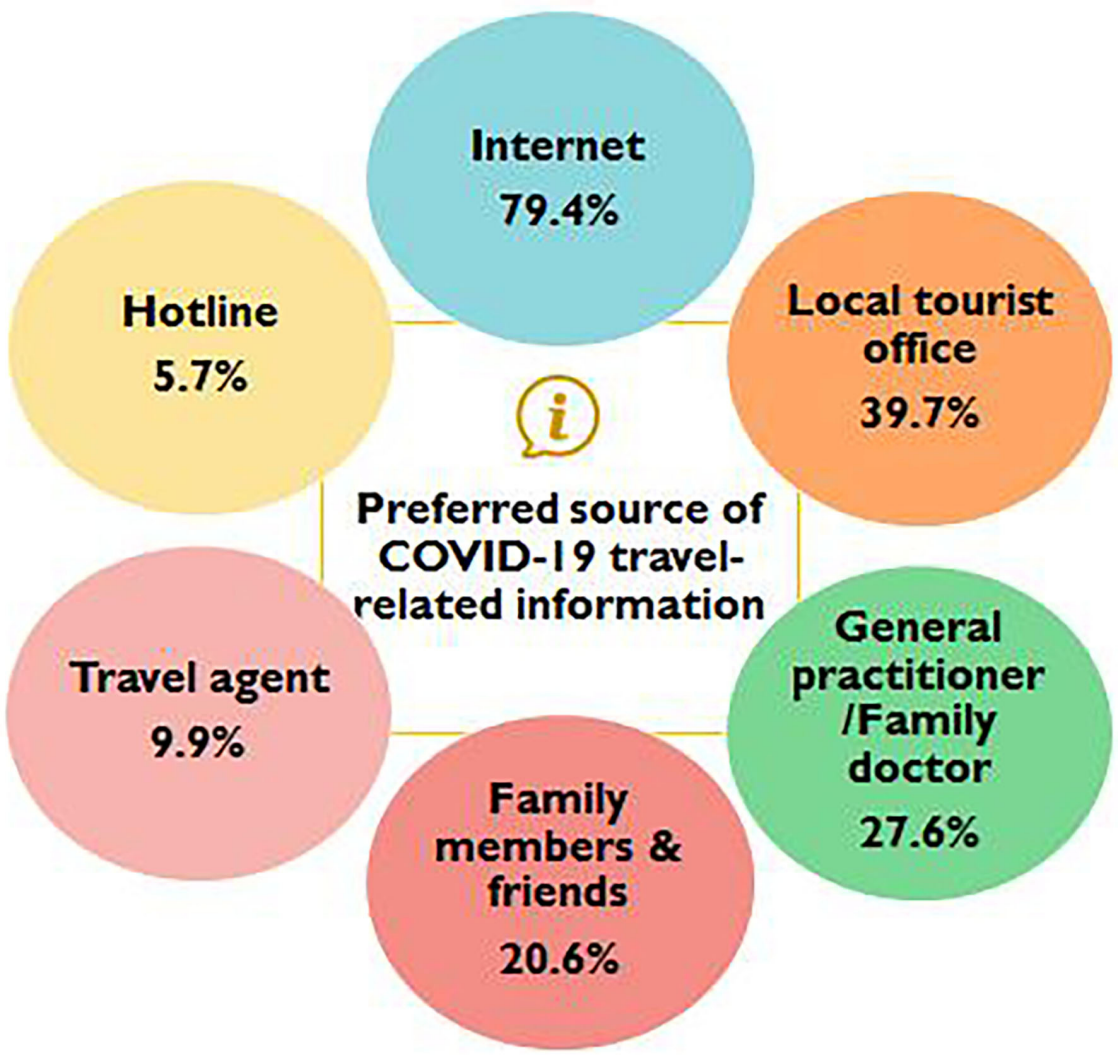
Preferred source of COVID-19 travel-related information (*N* = 3,237).

Figure 2 shows the findings of the preferred sources of COVID-19 travel-related information. The majority of the study participants preferred to refer to the Internet (79.4%) as their source of information, followed by the local tourist office (39.7%), and general practitioner/family doctor (27.6%).

## Discussion

One of the large under-researched aspects of the epidemiology of COVID-19 is how the public perceives the threat and modifies their behaviour during the massive outbreak. We report travel risk perceptions and intentions and their associated factors during the periods where COVID-19 is an ongoing nationwide emergency. Insights from this study can provide public health practitioners and policymakers with real-time situation awareness that can shape travel-related communication and intervention practises.

Travel to countries with an apparent community spread is an important contributor to the spread of the disease during an outbreak. Findings showed that the majority of the participants in this study have a reasonably high-risk perception of travel threats. On a positive note, only a small proportion had an inaccurate risk perception that only older adults and people with chronic medical conditions should consider postponing international travel and that if the recommended measures are practised, one will not get infected. As of today, scientists worldwide are still battling to understand the mechanisms of the transmission of the SARS-CoV-2 virus. Analysis of data related to the spread of SARS-CoV-2 in China seems to indicate that both respiratory droplets and aerosol transmission have been reported possible in the case of protracted exposure to elevated aerosol concentrations in closed spaces (19). The complete mechanism concerning COVID-19 transmission is not fully understood and is currently unclear (19); the public needs to take precautions and avoid any form of travel whenever possible.

Preliminary data suggest that older adults and persons with underlying health conditions or compromised immune systems might be at greater risk for severe illness from the SARS-CoV-virus (20, 21). In this study, risk perceptions were lower with regard to domestic travels, where a higher proportion reported practising recommended precautionary measures during domestic travel and the perception that only older adults and people with chronic medical condition should restrict travels. Of important note is that the application of non-pharmaceutical interventions (NPIs), which include social distancing, quarantine, isolation, school and workplace closure, and travel restrictions may limit the spread of disease (22). There have been suggestions that social distancing intervention should be continued for the next few months to prevent case numbers increasing again should lockdowns and travel restrictions be lifted. As such, limiting domestic travel is also an important NPI for the pandemic containment. The public need to be enlightened that even domestic travellers are exposed to nearly all the infectious risks of their travel destinations, as well as during the course of the travel itself.

Another key challenge in containment measures in terms of travel restrictions is finding the balance between the benefits and harms in travelling. In this study, the majority of participants that expressed intention for overseas and domestic travel noted the reasons for travel were non-essential, such as for leisure and holidays, with a minority associating their travel as being essential, such as work-related or for educational purposes. The public should be advised to avoid non-essential travel. Limited interactions, such as adjusting to working from home and supporting distance or e-learning should be encouraged during the COVID-19 outbreak.

Psychological functioning is known to be an important determinant of health outcomes. During the SARS outbreak, a moderate level of anxiety influenced precautionary measures against the infection (23). Nonetheless, in this study, the level of anxiety is not a significant influence on travelling intention. This is perhaps due to the successful containment of the spread of the coronavirus in Taiwan and the public are expressing a lower level of anxiety over the outbreak that has sparked global concern and fear. Taiwan is situated near Japan, mainland China and South Korea, three countries with some of the world's most severe outbreaks, but the island itself has just 48 isolated cases and one death. Of note, this study found higher anxiety levels among people who perceived their health status as poor, females and the lower-income groups, which will provide important information for interventions to reduce anxiety.

In this study, multivariable analyses revealed that risk perceptions were depicted as a strong influence in risk-taking international travel intentions. It is important to educate the public that the risk of COVID-19 infection is high for everyone, despite the evidence suggesting that the coronavirus is affecting older people and people with existing health conditions, who appear to be more vulnerable to becoming severely ill with the virus. Furthermore, as preventing and containing the spread of diseases through travel is extremely challenging, in the event where travel is unavoidable, the public is advised to strictly adhere to the recommendations for travellers, such as using a mask, maintaining a distance from others during travel and self-isolation upon returning from countries with apparent community spread (24).

The majority of study participants reported a preference for utilising the Internet for learning and finding information on COVID-19 travel-related information, implying the need to inform the public of the reliance on information from trusted sources. According to previous study, people often refer to travel agents for advice, including both their health risks and preventive actions they should be taking during trips (25), likewise found in this study. As such it would be useful if travel agencies to be provided with accurate information in order for them to contribute to the promotion of safe travel measures to the public (26).

The current study has several limitations. The first limitation is that the responses were based on self-reporting of data and may be subject to self-reporting bias and a tendency to report socially desirable responses. Secondly, the Internet-based questionnaire may have introduced selection bias. It is notable that the study has an over-representation of female participants. Nevertheless, during the period of crisis for the outbreak, the Internet-based questionnaire survey method, which used LINE and Facebook, the largest social media platform in Taiwan, was extremely effective in achieving a broad coverage of participants. Despite these limitations, the study contributes tremendously to the understanding of the psychological well-being and psycho-behavioural responses of the general public in Taiwan associated with COVID-19 while the epidemic is still ongoing.

## Conclusion

The present study provides important information on risk perceptions, and travel behaviours among the public in Taiwan during COVID-19 epidemic. In view of the coronavirus being highly pathogenic and extremely contagious, even a small proportion of the public expressing an intention to travel may lead to devastating consequences in terms of the spread of COVID-19 to the community. There is a need to limit human-to-human transmission, whereby limiting travel, particularly non-essential travel, is an important means. Our results imply that both domestic and international travel intention is prevalent during the early stage of the COVID-19 pandemic. Hence, there is a need for timely and immediate action to establish policy for enforcement of travel restrictions and border control measures to effectively limit transmission during the early phase of an outbreak. As the COVID-19 pandemic has impacted the tourism industries and the general well-being of the public in unprecedented ways, tourism stakeholders should embark on discussion and research about converting this crisis disruption into transformative innovation, such as e-tourism, to promote sustainable tourism throughout the pandemic. It is also timely that the government establish travel bubbles with other countries to facilitate the recovery of the tourism sector and reduce the COVID-19 importation.

## Data Availability Statement

The raw data supporting the conclusions of this article will be made available by the authors, without undue reservation.

## Ethics Statement

This study protocol was approved by the Institutional Review Board of National Cheng Kung University Hospital (NCKUH) (No: A-EX-109-013). Participants were informed that their participation was voluntary, and consent was implied upon completion of the questionnaire. All responses were collected and analysed without identifiers.

## Author Contributions

LPW and C-JC conceived the study. C-JC conducted and collected data. LPW and HA analysed the data. LPW, YL, ZH, and TS-HL wrote the manuscript. All authors have approved the manuscript.

## Conflict of Interest

The authors declare that the research was conducted in the absence of any commercial or financial relationships that could be construed as a potential conflict of interest.

## Publisher's Note

All claims expressed in this article are solely those of the authors and do not necessarily represent those of their affiliated organizations, or those of the publisher, the editors and the reviewers. Any product that may be evaluated in this article, or claim that may be made by its manufacturer, is not guaranteed or endorsed by the publisher.
